# Vaccine-Induced IgG Antibodies to V1V2 Regions of Multiple HIV-1 Subtypes Correlate with Decreased Risk of HIV-1 Infection

**DOI:** 10.1371/journal.pone.0087572

**Published:** 2014-02-04

**Authors:** Susan Zolla-Pazner, Allan deCamp, Peter B. Gilbert, Constance Williams, Nicole L. Yates, William T. Williams, Robert Howington, Youyi Fong, Daryl E. Morris, Kelly A. Soderberg, Carmela Irene, Charles Reichman, Abraham Pinter, Robert Parks, Punnee Pitisuttithum, Jaranit Kaewkungwal, Supachai Rerks-Ngarm, Sorachai Nitayaphan, Charla Andrews, Robert J. O’Connell, Zhi-yong Yang, Gary J. Nabel, Jerome H. Kim, Nelson L. Michael, David C. Montefiori, Hua-Xin Liao, Barton F. Haynes, Georgia D. Tomaras

**Affiliations:** 1 Department of Veterans Affairs New York Harbor Healthcare System, New York, New York, United States of America; 2 New York University School of Medicine, New York, New York, United States of America; 3 Vaccine and Infectious Disease Division, Fred Hutchinson Cancer Research Center, Seattle, Washington, United States of America; 4 Duke University, Durham, North Carolina, United States of America; 5 Public Health Research Institute, University of Medicine and Dentistry, Newark, New Jersey, United States of America; 6 Faculty of Tropical Medicine, Mahidol, Thailand; 7 Ministry of Public Health, Bangkok, Thailand; 8 Armed Forces Research Institute of Medical Sciences, Bangkok, Thailand; 9 Military HIV Research Program, Walter Reed Army Institute of Research, Silver Spring, Maryland, United States of America; 10 Virology Laboratory, Vaccine Research Center, National Institute for Allergy and Infectious Diseases, National Institutes of Health, Bethesda, Maryland, United States of America; University of Pittsburgh, United States of America

## Abstract

In the RV144 HIV-1 vaccine efficacy trial, IgG antibody (Ab) binding levels to variable regions 1 and 2 (V1V2) of the HIV-1 envelope glycoprotein gp120 were an inverse correlate of risk of HIV-1 infection. To determine if V1V2-specific Abs cross-react with V1V2 from different HIV-1 subtypes, if the nature of the V1V2 antigen used to asses cross-reactivity influenced infection risk, and to identify immune assays for upcoming HIV-1 vaccine efficacy trials, new V1V2-scaffold antigens were designed and tested. Protein scaffold antigens carrying the V1V2 regions from HIV-1 subtypes A, B, C, D or CRF01_AE were assayed in pilot studies, and six were selected to assess cross-reactive Abs in the plasma from the original RV144 case-control cohort (41 infected vaccinees, 205 frequency-matched uninfected vaccinees, and 40 placebo recipients) using ELISA and a binding Ab multiplex assay. IgG levels to these antigens were assessed as correlates of risk in vaccine recipients using weighted logistic regression models. Levels of Abs reactive with subtype A, B, C and CRF01_AE V1V2-scaffold antigens were all significant inverse correlates of risk (p-values of 0.0008–0.05; estimated odds ratios of 0.53–0.68 per 1 standard deviation increase). Thus, levels of vaccine-induced IgG Abs recognizing V1V2 regions from multiple HIV-1 subtypes, and presented on different scaffolds, constitute inverse correlates of risk for HIV-1 infection in the RV144 vaccine trial. The V1V2 antigens provide a link between RV144 and upcoming HIV-1 vaccine trials, and identify reagents and methods for evaluating V1V2 Abs as possible correlates of protection against HIV-1 infection.

**Trial Registration:**

ClinicalTrials.gov NCT00223080

## Introduction

The RV144 HIV-1 clinical vaccine trial using ALVAC-HIV and AIDSVAX gp120 B/E resulted in an estimated vaccine efficacy of 31.2% measured 36 months after the six month vaccination series [1]. The level of IgG antibodies (Abs) binding to a fusion protein consisting of the first and second variable regions (V1V2) of an HIV-1 gp120 envelope glycoprotein and the gp70 of murine leukemia virus [2] was identified as a statistically significant inverse correlate of risk (CoR) of HIV-1 infection [3], [4].

The identification of an inverse CoR with Ab reactivity to a variable region of the HIV-1 gp120 envelope protein provided support for the hypothesis that Abs that bind to envelope variable regions may interfere with infection [2], [5]–[8]. Since 89% of the infections occurring in the RV144 vaccine trial were caused by HIV-1 CRF01_AE (subtype AE) and the V1V2 IgG CoR was with a fusion protein carrying the V1V2 region from a subtype B strain [3], we hypothesized that the protective Abs in RV144 were cross-reactive with the V1V2 regions found in viruses from many HIV-1 subtypes. To clarify the extent of V1V2 cross-reactive Abs associated with decreased risk of infection, to determine if comparable findings could be generated using a different assay, and to identify reagents for evaluation of CoRs in future efficacy trials, new V1V2 antigens were designed and tested for their ability to predict decreased risk of HIV-1 transmission in the RV144 trial.

## Methods

### Ethics Statement

The RV144 clinical vaccine trial was registered with ClincialTrials.gov and assigned a registration number of NCT00223080. The protocol for this trial is available as supporting information; see Protocol S1. It was approved by all relevant institutional and governmental committees, and the protocol of the trial was described in Rerks-Ngarm et al. [1]. Specifically, the institutional review boards of the Thai Ministry of Public Health Ethics Committee, the Royal Thai Army Medical Department, the Ethics Committee of the Faculty of Tropical Medicine, Mahidol University, and the US Surgeon General’s Human Subjects Research Review Board approved the protocol and attendant immune correlates work. All subjects provided written informed consent and passed a test of understanding as previously described [1]. Briefly, RV144 was a community-based, randomized, multicenter, double-blind, placebo-controlled vaccine efficacy trial consisting of four injections of a recombinant canarypox vector vaccine (ALVAC-HIV [vCP1521]) given at 0, 1, 3, and 6 months, and two injections of recombinant gp120 subunits (AIDSVAX B/E®) given at months 3 and 6. The vaccine and placebo injections were administered to 16,402 healthy men and women between the ages of 18 and 30 years in Thailand. Enrollment screening began on 24 Sep 2003, and the study ended 30 June, 2009.

*Specimens used.* The assessment of Ab reactivity of RV144 vaccine and placebo recipients was performed with three panels of plasma. In the initial “Phase 1” pilot study, 32 uninfected vaccinees’ plasma were used (Set C), drawn at Week 26, corresponding to two weeks after the last administered dose. In a subsequent study (“Phase 2”), a plasma panel (Set V2L) was used consisting of plasma specimens from 40 uninfected vaccinees and 20 uninfected placebo recipients drawn at Weeks 0 and 26. The ensuing study focused on Week 0 and 26 specimens selected for the case-control study [3] which consisted of 41 infected vaccinees, 205 frequency-matched uninfected vaccinees, 20 infected and 20 uninfected placebo recipients.

### Reagents and Assays

**Figure 1:**
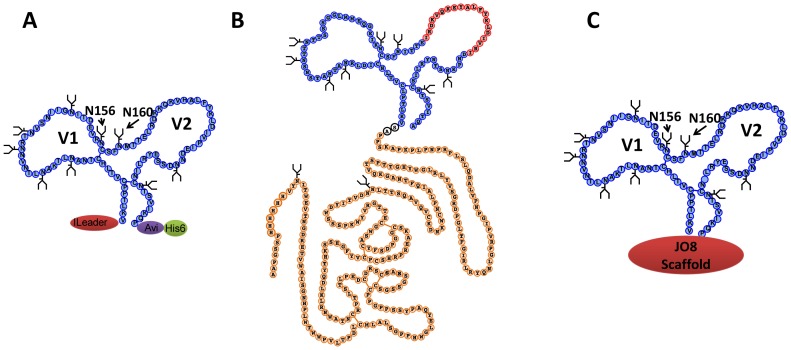
Diagrams of the V1V2-scaffold antigens used to study V1V2 antibodies in RV144 plasma specimens. (A) tags-V1V2 scaffold antigen [9]; (B) gp70-V1V2 scaffold antigen [2]; (C) JO8-V1V2 scaffold antigen. [10], [11].

To test for Abs present in the plasma of RV144 participants with activity specific for epitopes in the V1V2 region of the HIV-1 gp120 envelope glycoprotein, a total of 22 V1V2-scaffold antigens were constructed carrying V1V2 sequences from HIV-1 subtypes A, B, C, D or CRF01_AE (subtype AE) expressed in the context of three scaffolds [2], [9]–[11]. The scaffold antigens used are listed in **Table 1** and diagramed in **Figure 1**. The sequences of the V2 segments in the V1V2-scaffold antigens used for the case-control study are listed in **Table 2**. The V1V2 sequence of subtype B strain CaseA2 in both plasmids 565f pur and p623 (**Table 1**) are the same as that listed for gp70.B(CaseA2.p623)-V1V2.AP_orig_.

**Table 1:** V1V2 scaffold antigens used to study the reactivity of plasma specimens from RV144 vaccine and placebo recipients.

V1V2-scaffold Antigens	Phase 1+	Phase 2‡	Case-Control	
			ELISA*	BAMA**
gp70_WT_.AP		1∶100§		
gp70_WT_.LL	+			
gp70.B(Case A2.p623)-V1V2.AP_orig_		1∶100	1∶100	1∶100
gp70.B(Case A2.p623)-V1V2.AP_(new batch)_		1∶100		
J08.C(97ZA012)-V1V2	+			
J08.B(HxB2)-V1V2	+			
J08.A(92RW020)-V1V2	+			
tags.B(Case A2)-V1V2	+			
tags.B(63521)-V1V2	+			
gp70.B(Case A2)-V1V2.LL	+	1∶100	1∶100	1∶100
gp70.A(Q23)-V1V2	+			
gp70.C(97ZA012)-V1V2	+	1∶100	1∶100	1∶100
gp70.B(Case A2)-V1V2		1∶100		
gp70.B(HxB2)-V1V2	+	1∶100		
gp70.B(Case A2.p565f pur)-V1V2	+	1∶100		
tags.A(Q23)-V1V2	+	1∶300		
gp70.A(92RW020)-V1V2	+	1∶100	1∶100	1∶100
tags.A(9004SS)-V1V2	+	1∶100		
tags.D(9009SA)-V1V2	+			
tags.AE(92TH023)-V1V2	+			
tags.AE(A244/D7N)-V1V2	+			
tags.C(1086)-V1V2	+	1∶300	1∶300	1∶100
tags.AE(A244)-V1V2	+	1∶100		
gp70.AE(92TH023)-V1V2	+	1∶900	1∶900	1∶100

+ indicates six 3-fold serial plasma dilutions starting with 1∶100 used in Phase 1.

‡ indicates plasma dilution used in Phase 2 and case-control experiments.

* ELISA = enzyme-linked immunosorbent assay;

** BAMA = binding antibody multiplex assay;

§ Plasma dilutions used in assays.

**Table 2:** V2 sequences in V1V2-scaffold antigens tested with case-control specimens*.

V1V2-scafffold Antigens	V2 Sequences
**gp70.B(CaseA2)-V1V2.AP_orig_**	CSFNITTS**IRDKVQKEYALFYK** LDIVPI.DNPKNST.N.YRLISC
**gp70.B(CaseA2)-V1V2.LL**	CSFNITTS**IRDKVQKEYALFYK** LDIVPI.DNPKNST.N.YRLISC
**gp70.C(97ZA012)-V1V2**	CSFNTTTE**IRDKKQQGYALFYR** PDIVLLKENRNNSNNSEYILINC
**gp70.AE(92TH023)-V1V2** ‡	CSFNMTTE**LRDKKQKVHALFYK** LDIVPIEDNTSSS.E.YRLINC
**tags.C.(1086)-V1V2**	CSFKATTE**LKDKKHKVHALFYK** LDVVPL.NGNSSSSGE.YRLINC
**gp70.A(92RW020)-V1V2**	CSFNITTE**LKDKKQQVYSLFYK** LDVVQINEKNET.D.K.YRLINC

* Sequence of V2 residues present in the designated V1V2-scaffold antigens. Residues in bold indicate the V2 epitope recognized by RV144 vaccinees’ plasma as mapped with peptides [4]. Underlined residues represent the putative α4β7 binding site.

‡ Sequence of V1V2 in ALVAC-HIV subtype E gp120 priming immunogen.

Gp70-V1V2 scaffold antigens provided by Dr. A. Pinter were made as follows: gp70.B(CaseA2/p565f pur)-V1V2 and gp70.AE (92TH023)-V1V2 fusion proteins were expressed by joining V1V2 residues 120–204 (HxB2 numbering system) to the C-terminus of an N-terminal fragment of the Friend MuLV gp70 protein (residues 1–263, with a His6 signal inserted at position 9) and purified by chromatography on Ni-NTA beads, as described previously [2]. The gp70.B(CaseA2/p623)-V1V2 antigen contained a TEV protease cleavage site (ENLYFQSAS) inserted between the gp70 and V1V2 sequence. A wild type control gp70_WT_ (consisting of gp70 amino acids1–263) was also prepared and purified in the same manner. The sequence of the gp70 carrier protein is: SAAPGSSPHHHHHHVYNITWEVTNGDRETVWAISGNHPLWTWWPVLTPDLCMLALSGPPHWGLEYQAPYSSPPGPPCCSGSSGSSAGCSRDCDEPLTSLTPRCNTAWNRLKLDQVTHKSSEGFYVCPGSHRPREAKSCGGPDSFYCASWGCETTGRVYWKPSSSWDYITVDNNLTTSQAVQVCKDNKWCNPLAIQFTNAGKQVTSWTTGHYWGLRLYVSGRDPGLTFGIRLRYQNLGPRVPIGPNPVLADQLSLPRPNPLPKPAKSPPAS.

V1V2 scaffold antigens using the JO8 and tags scaffolds were made as described in Liao et al. [9] The tags scaffold antigens, tags.A(Q23)-V1V2, tags.A(9004SS)-V1V2, tags.D(9009SA)-V1V2, tags.AE(A244)-V1V2, tags.AE(92TH023)-V1V2 and tags.C(1086)-V1V2, were made with an N-terminal Ig VH leader sequence, and an Avi tag and His6 tag at the C-terminal end for labeling and purification, respectively (see **Figure 1**). Seven of nine N-glycosylation sites (except two at positions 156 and 160) in tags.AE(A244)-V1V2 were abolished resulting in a new construct, tags.AE(A244/D7N)-V1V2, by changing the asparagine residues to aspartic acid residues. gp70.B(CaseA2)-V1V2.LL and gp70.A(92RW020)-V1V2 were made as fusion proteins with MuLV gp70 and produced as described above and by Pinter et al. [2] All V2 gene constructs were codon-optimized by converting amino acid sequences to nucleotide sequences employing the codon usage of highly expressed human housekeeping genes [12]. The recombinant V1V2 scaffold antigens were produced in 293F cells by transient transfection, purified by using nickel columns and stored at −80°C until use.

### Immunologic Assays

The presence of reactive Abs was assessed using enzyme-linked immunosorbent assay (ELISA) and a binding Ab multiplex assay (BAMA) [13] performed in independent laboratories.

### ELISA Assays

The initial Phase 1 antigenicity study using plasma set C and V1V2-scaffold antigens utilized a direct binding ELISA conducted in 384 well ELISA plates coated with 2 ug/ml antigen in 0.1 M sodium bicarbonate and blocked with assay diluent (PBS containing 4% (w/v) whey protein, 15% normal goat serum, 0.5% Tween-20, and 0.05% sodium azide). Plasma were incubated for 90 min in three-fold serial dilutions beginning at 1∶30, followed by washing with PBS with 0.1% Tween-20. Ten microliters of HRP-conjugated goat anti-human IgG was diluted to 1∶10,000 in assay diluent without azide, incubated for 1 hour, washed, and color was developed with 20 µl of SureBlue Reserve for 15 minutes. The reaction was stopped with the addition of 20 ul HCl stop solution. Plates were read at 450 nm. For these data, baseline levels were significant and therefore were subtracted from all values. Results generated with this method are shown as “baseline-adjusted”.

The second down-selection experiment (Phase 2) was performed with plasma Set V2L. This experiment, as well as the studies of the case-control plasma panel, was conducted using an ELISA method previously described [3]. Briefly, StreptaWell plates were coated with 1 µg/ml V1V2-scaffold antigens or with unmodified gp70 (gp70_WT_) for 1.5 h at 37°C and then washed six times with PBS containing 0.05% Tween-20, before incubation for 1.5 h at 37°C with plasma diluted in RPMI media containing 15% fetal bovine serum. The plates were washed six times, and alkaline phosphatase-conjugated goat anti-human IgG (1∶2000) was added for 1.5 h at 37°C. After washing, 10% diethanolamine substrate was added for 30 min to develop color, and the plates were read at 405 nm. At each step, every well contained 50 µl; specimens were run in duplicate in each experiment, and three experiments were performed. Baseline-adjusted read-outs were not used for expressing the results in these assays since this was not done in the original correlates analysis and the baseline read-outs were low, particularly as compared to the read-outs generated in the ELISA method used in the initial (Phase 1) pilot study.

### Binding Antibody Multiplex Assay (BAMA)

This assay was performed because it is a high through-put, standardized assay which can be run efficiently with multiple ligands, making it an ideal assay for future vaccine studies. Plasma HIV-1 specific antibodies to V1V2 scaffolds were measured by a custom HIV-1 BAMA as previously described [3], [13]. All assays were run under GCLP compliant conditions, including tracking of positive controls by Levy-Jennings charts using 21CFR Part 11 compliant software. Positive controls included titration of a HIVIG preparation and V2-specific monoclonal Ab (mAb) CH58 [9]. Negative controls included in every assay were blank beads, HIV-1 negative sera, and baseline (pre-vaccination) samples. To control for antigen performance, we used the pre-set criteria that the positive control titer (HIVIG) included in each assay (and for assays with V1V2 antigens, CH58 mAb) had to be within +/−3 standard deviations of the mean for each, with pre-set acceptance of titer (calculated with a four-parameter logistic equation, SigmaPlot, Systat Software). Antibody measurements were acquired on a Bio-Plex instrument (Bio-Rad, Hercules, CA), and the read-out was as mean fluorescent intensity (MFI). The pre-set assay criteria for sample reporting were coefficient of variation per duplicate values of <15% for each sample and >100 beads counted per sample.

### Statistical Analysis

Applying the approach used previously [3], weighted logistic regression models were used to assess levels of IgG V1V2-scaffold-specific Abs measured at Week 26 as CoRs of the outcome HIV-1 infection in vaccine recipients, controlling for gender and baseline behavioral risk, with plasma IgA HIV-1 Env binding Abs included as a quantitative variable. This IgA variable was the same as the primary IgA variable assessed in the original analysis [3], and was controlled for because it significantly and independently predicted HIV-1 infection risk. Because the control:case ratio (205∶41; i.e., 5∶1) of subjects with IgG and IgA measured was less than the ratio of all controls and all cases at-risk at Week 26 (7010∶41; i.e., 171∶1), use of ordinary logistic regression would provide a biased assessment. To correct this bias, the model weights each subject by the reciprocal of the estimated probability that s/he had IgG and IgA measured and therefore was included in the statistical analysis; these estimated probabilities were computed as observed fractions of vaccinees with IgG and IgA data divided by all at-risk vaccinees within each stratum defined by the cross-classification of case status, gender, per-protocol status, and the number of immunizations received.

The IgG immune variables were modeled quantitatively, as well as using Low/Medium/High categories based on tertiles of response in the vaccine group. Individual IgG variables were assessed as CoRs in separate models, and pairs of IgG variables with Spearman rank correlation below 0.90 were assessed as CoRs in multivariable models. (These models include five independent variables: the two IgG variables, the IgA variable, gender, and baseline behavioral risk.) Four immune score variables measuring cross-reactivity were also assessed as CoRs; these scores are defined in **File S1**. The analyses were conducted separately for ELISA and BAMA. See **File S1** for additional details.

## Results

### V1V2-scaffold Antigens Studied

A total of 22 V1V2-scaffold constructs (**Table 1**) were used to study the V1V2-specific Ab responses of participants in the RV144 clinical vaccine trial. These included the gp70.B(CaseA2.p623)-V1V2.AP_orig_ reagent used in the initial case-control study (designated V1V2_orig_-gp70) [3], and two lots of the truncated form of the wild type murine leukemia virus gp70 as controls (gp70_WT_) [2]. The V1V2 sequences used to form the V1V2-scaffold antigens were derived from five HIV-1 subtypes (A, B, C, D, and AE). Three types of V1V2 constructs were employed: the original truncated form of V1V2 fused to MuLV gp70 [2], V1V2 inserted between an Ig leader sequence and aviden and HIS tags (“tags”) [9], and V1V2 scaffold on the JO8 protein [10], [11] (**Figure 1**).

### Initial Antigenic Assessment of V1V2-scaffolds

**Figure 2:**
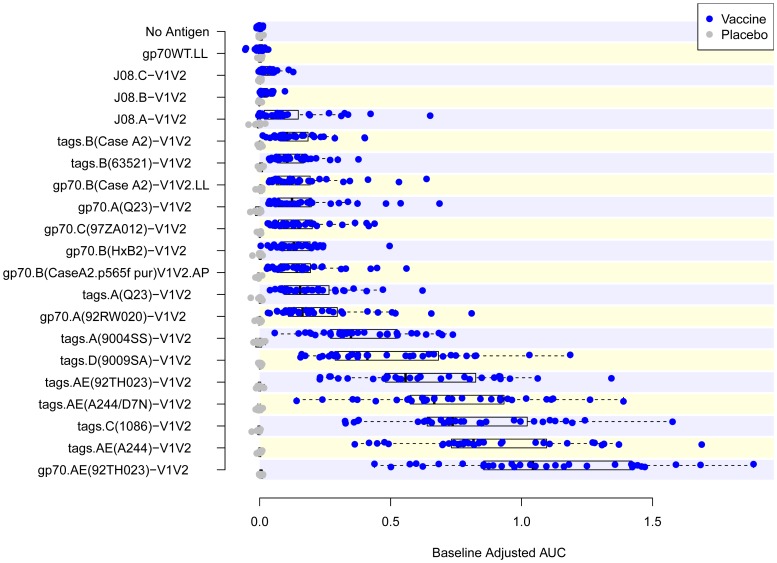
Reactivity in ELISA of the 19 V1V2-scaffold antigens used in the Phase 1 study. Results are shown as box plots of reactivity with each of the 19 Phase 1 V1V2-scaffold antigens and two negative controls (“No Antigen” and “gp70_WT_”) with 40 plasma samples from pilot sample set C. Results shown are the baseline-adjusted (i.e., Week 26 minus Week 0) areas under the dilution curves (AUC).

**Figure 3:**
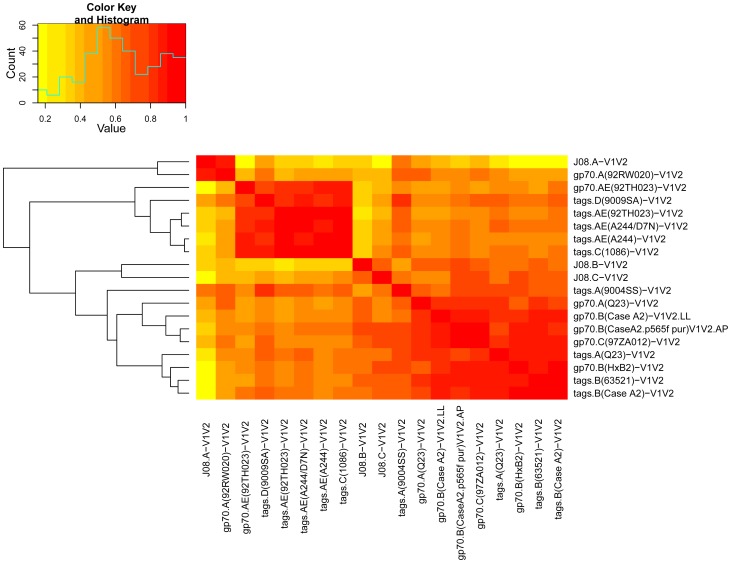
Heat map and hierarchical clustering tree of Spearman rank correlations of pairs of 19 V1V2-scaffold antigens. The heat map and clustering tree are based on ELISA results derived from Phase 1 studies with specimens from 32 vaccine recipients from pilot sample set C using the baseline adjusted (i.e., Week 26 minus Week 0) area under the dilution curve read-out.

In order to test the antigenicity and select a subset of V1V2-scaffolds that would include reagents with various immunologic characteristics, 19 V1V2-scaffolds and gp70_WT_.AP were tested by ELISA in an initial “Phase 1” study (**Table 1**) using a set of plasma specimens drawn before immunization (Week 0) and two weeks after the last immunization (Week 26) from eight placebo and 32 vaccine recipients. **Figure 2** shows the reactivity in ELISA of plasma from recipients of the vaccine or the placebo against each of the antigens. Comparison of the reactivities of plasma from each group drawn at Week 26 shows a response to all of the 19 V1V2-scaffolds tested with considerable variability across antigens. A heat map was generated from these data using Spearman rank correlation values between all pairs of reagents (**Figure 3**). Based on the response rates and levels of reactivity, reproducibility of read-outs across within-subject replicates, and similarities and differences in reactivity patterns between the various V1V2-scaffolds, 10 of the 19 V1V2-scaffold antigens were included in a second “Phase 2” analysis to further down-select the V1V2-scaffolds for the final set of antigens to be run with case-control specimens.

**Figure 4:**
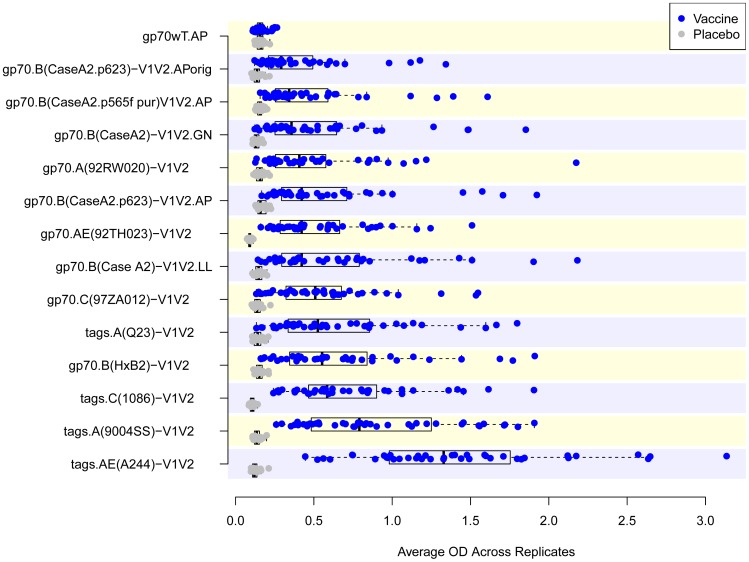
Reactivity in ELISA of the 13 V1V2-scaffold antigens used in the Phase 2 study. Results are shown as box plots of reactivity with each of the 13 Phase 2 V1V2-scaffold antigens and the scaffold control antigen (gp70_WT_) with 60 plasma samples from set V2L. Results shown are Week 26 read-outs at a 1∶100 dilution with the exception of tags.A(Q23)-V1V2 and tags.C(1086)-V1V2 which were run at a 1∶300, dilution and gp70.AE(92TH023)-V1V2 which was run at 1∶900. AP, LL and GN denote production of comparable reagents by Drs. Pinter, Liao, and Nabel.

**Figure 5:**
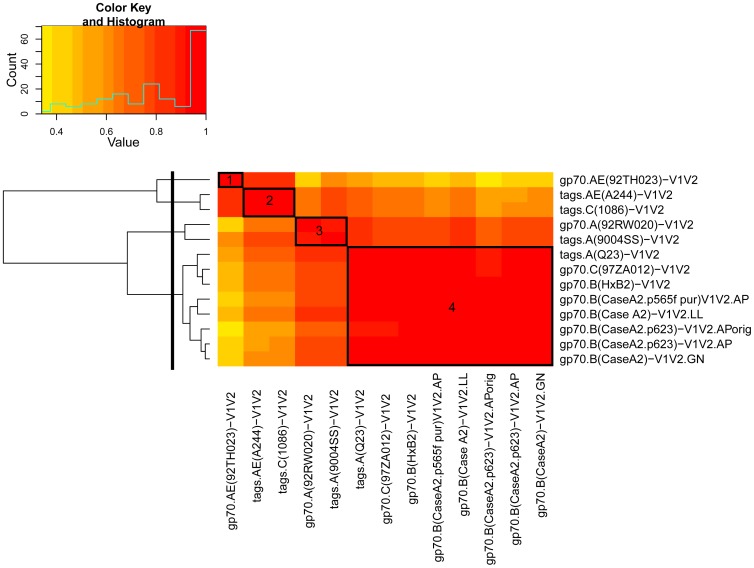
Heat map and hierarchical clustering tree of Spearman rank correlations of pairs of 13 V1V2-scaffold antigens. The heat map and hierarchical clustering tree are derived from ELISA data from all pairs of the 13 Phase 2 V1V2-scaffold antigens, and are based on read-outs from the 40 vaccine recipients from pilot sample set V2L. The solid black line to the left cuts the clustering tree into four clusters. The four boxes, labeled 1–4, highlight the pairwise correlations of each of these clusters. AP, LL and GN denote production of comparable reagents by Drs. Pinter, Liao and Nabel.

For the second antigen down-selection, plasma Set V2L was tested by ELISA for reactivity with gp70_WT_.AP and 13 V1V2-scaffolds (**Table 1**). In addition to the 10 V1V2-scaffolds chosen from the Phase 1 down-selection experiments, the original batch of V1V2_orig_-gp70 was used along with a new batch made by the same lab with the same plasmid, and another batch made independently in a different lab. **Figure 4** shows that all V1V2-scaffold antigens detect a vaccine-induced response, with a highly significant difference in magnitude comparing read-outs between vaccine and placebo recipients. The response rates ranged from a low of 68% for gp70.A(92RW020)-V1V2 to 100% for several reagents (**Table 3**). A cluster analysis of the reactivities was performed to identify which reagents were most closely related antigenically. Four clusters were identified as depicted in the heat map shown in **Figure 5** and **Table 3**. For the study with the case-control specimens, six V1V2-scaffold antigens were chosen: from the 13 tested in the Phase 2 experiment: one each from clusters 1–3 and three from cluster 4 (**Figure 5** and **Table 3**); these included reagents carrying V1V2 sequences from subtypes A, B, C and AE, displayed on the gp70 and “tags” scaffolds (see **Figure 1** **)**. One of these reagents was the same V1V2 antigen, gp70.B(CaseA2.p623)-V1V2.AP_orig_, used in the initial case-control study (designated hereinafter as V1V2_orig_-gp70) [3]. The V2 sequences included in each of the V1V2-scaffold antigens used in the case-control study are shown in **Table 2**.

**Table 3:** Data used for down-selection of V1V2-scaffold antigens to be used in the case-control studies.

Cluster	Dilution	Correlation*	Response§	Signal-to-noise§	P-value§
**1**					
**gp70.AE(92TH023)-V1V2**	1∶900	0.39	40/40 = 100%	4.55	2e-12
**2**					
tags.AE(A244)-V1V2	1∶100	0.56	40/40 = 100%	3.66	2e-12
**tags.C(1086)-V1V2** ‡	1∶300	0.57	40/40 = 100%	4.26	2e-12
**3**					
**gp70.A(92RW020)-V1V2**	1∶100	0.77	27/40 = 68%	13.39	2e-10
tags.A(9004SS)-V1V2	1∶100	0.76	40/40 = 100%	6.97	4e-08
**4**					
tags.A(Q23)-V1V2	1∶300	0.94	34/40 = 85%	4.19	5e-12
**gp70.C(97ZA012)-V1V2**	1∶100	0.95	32/40 = 80%	4.95	3e-11
gp70.B(HxB2)-V1V2	1∶100	0.97	32/40 = 80%	9.91	8e-08
gp70.B(CaseA2.p565f pur)V1V2	1∶100	0.97	34/40 = 85%	4.58	4e-08
**gp70.B(Case A2)-V1V2.LL**	1∶100	0.97	36/40 = 90%	10.42	4e-12
**gp70.B(CaseA2.p623)-V1V2.APorig**	1∶100	0.98	NA	5.50	NA
gp70.B(CaseA2.p623)-V1V2	1∶100	1.00	32/40 = 80%	6.76	3e-11
gp70.B(CaseA2)-V1V2	1∶100	0.99	32/40 = 80%	6.57	2e-11

* Correlation: the Spearman correlation between the original gp70-V1V2 antigen read-out and the listed scaffold antigen.

§ Response, signal-to-noise, and P-values are defined in the Methods section.

‡ Antigens shown in bold were selected for use in case-control study.

**Figure 6:**
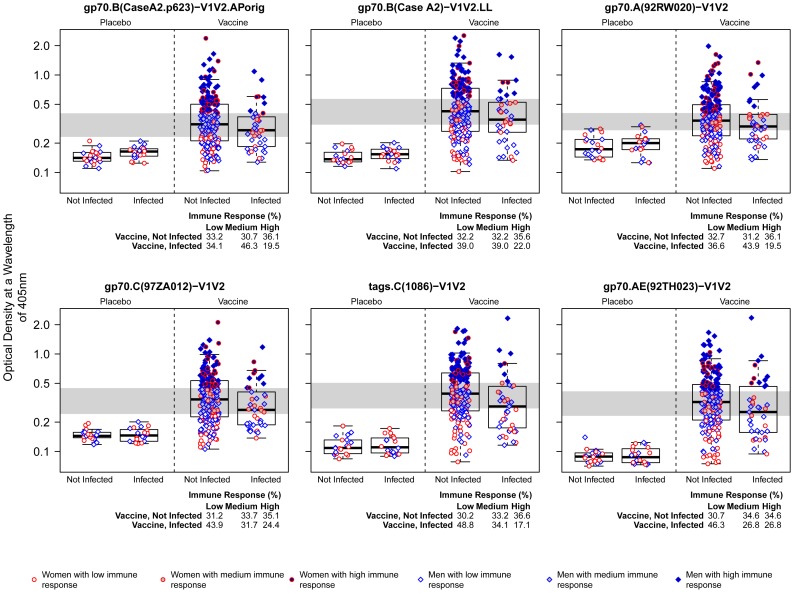
Reactivity in ELISA of V1V2-scaffold antigens with plasma from Week 26 case-control specimens. Results are shown as box plots of reactivity with each of the six V1V2-scaffold antigens with plasma from the 286 case-control specimens. Values are from ELISA-generated data with optical density (OD) read-outs based on a wavelength of 405 nM. Data were generated using plasma diluted 1∶100, except for assay of gp70.AE(92TH023)-V1V2 (1∶900) and tags.C(1086)-V1V2 (1∶300). Box plots show the 25^th^ percentile (lower edge of the box), 50^th^ percentile (horizontal line in the box), and 75^th^ percentile (upper edge of the box). Participants are stratified according to HIV-1 infection status and treatment assignment. Gender and immune response categories are indicated by the color and shape of the points. Low, Medium, and High are defined as tertiles of the Week 26 immune response for the vaccine group, and are divided by the gray shaded horizontal bands. AP and LL denote production of comparable reagents by Drs. Pinter and Liao.

**Figure 7:**
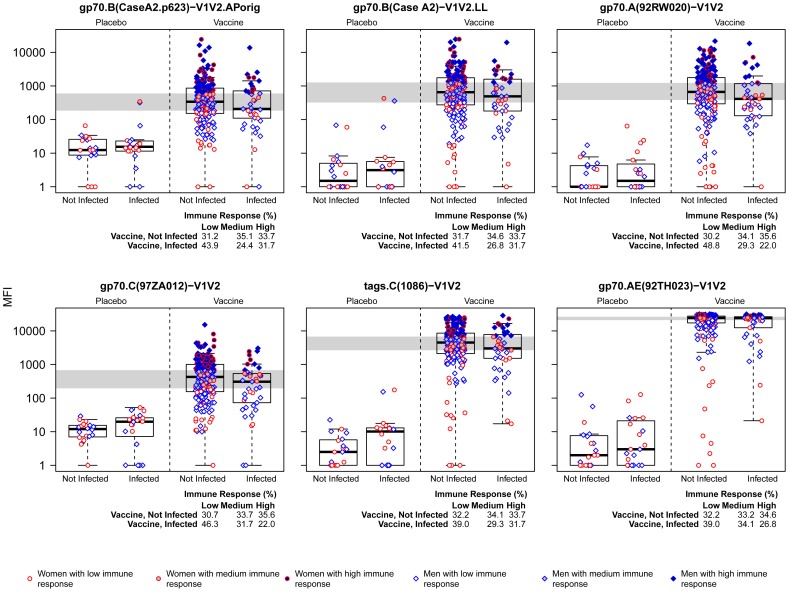
Reactivity in BAMA of V1V2-scaffold antigens with plasma from Week 26 case-control specimens. Results are shown as box plots of reactivity of each of the six V1V2-scaffold antigens with plasma from the 286 case-control specimens. All data were generated using plasma diluted 1∶100. The y-axis shows the natural log-transformation of the median fluorescence intensity (MFI).

The selected V1V2-scaffold antigens were then tested for reactivity by two different assays in independent laboratories using the original RV144 case-control specimen panel in which all specimens were coded, and analysis of the data was performed by an independent statistical team. As shown descriptively in **Figures 6** **and** **7**, whether tested for reactivity in ELISA or BAMA, there were higher responses against each of the six antigens in the uninfected vaccinees than in the infected vaccinees with each of the six V1V2-scaffold antigens; these responses are all significantly different based on the logistic regression analyses reported below and in **Table 4**. No reactivity was noted in placebo recipients by ELISA or BAMA; reactivity rates in vaccinees ranged from 48–96% depending on the antigen tested and method of analysis (**Table 5**). Plasma reactivity was within the dynamic range using the conditions described for ELISA, but reached saturation in BAMA for two of the six antigens tested (**Figure 7**). Nevertheless, in vaccine recipients, the level of reactivity with all six V1V2-scaffold antigens, run in both assays, yielded odds ratios (ORs) of HIV-1 infection (per 1-SD increment in antibody level) ranging from 0.53–0.68 and p-values ranging from 0.0008–0.05 (**Table 4**). The 286 plasma specimens from the case-control panel were run at a dilution of 1∶100 against all V1V2 scaffold antigens in BAMA. In ELISA, this same dilution was used except for tags.AE(A244)-V1V2.LL (1∶300) and gp70.AE(92TH023)-V1V2.AP (1∶900); this might have contributed to the differences noted between the two assays in terms of the relative order and values for the ORs of HIV-1 infection per 1-SD increment in Ab level and p-values. Nonetheless, the results of both ELISA and BAMA show the consistency of the inverse correlation with HIV infection risk across many V1V2 variants and across two independently run studies using different assays for V1V2 Abs.

**Table 4:** IgA-adjusted odds ratios of risk of HIV-1 infection (and p-values) based on data of Week 26 plasma reactivity with V1V2-scaffold antigens derived from two independent immunoassays.*

	ELISA‡		BAMA‡	
	OR	p-value	OR	p-value
gp70.B(Case A2)-V1V2.AP_orig_(primary)¥ §	0.63	0.0179	ND	ND
gp70.B(Case A2.p623)-V1V2.AP_orig_ †	0.62	0.0149	0.60	0.0059
gp70.B(Case A2)-V1V2.LL†	0.60	0.0084	0.59	0.0031
gp70.A(92RW020)-V1V2	0.68	0.0521	0.61	0.0071
gp70.C(97ZA012)-V1V2	0.56	0.0040	0.55	0.0008
tags.C(1086)-V1V2	0.53	0.0014	0.62	0.0039
gp70.AE(92TH023)-V1V2	0.62	0.0130	0.67	0.0097
Score (cross-reactivity)	0.58	0.0073	0.56	0.0015

* Odds ratios are for every one standard deviation increase in the natural log-transformed OD (ELISA) or MFI (BAMA) and p-values for testing odds ratios equal to one are calculated using IgA-adjusted weighted logistic regression models as described in the text. The cross-reactivity score measures the average log10 response level across the four subtypes represented in the 6 V1V2 scaffolds, as defined in File S1.

‡ ELISA = enzyme-linked immunosorbent assay; BAMA = binding antibody multiplex assay.

¥ Data published in the original case-control study [3].

† AP and LL denote production of comparable reagents by Drs. Pinter and Liao.

§ Reagent used in the original case-control study [3].

**Table 5:** Response rates in Placebo and Vaccine recipients as measured by ELISA and BAMA.

Scaffold-V1V2 Antigens	ELISA*	ELISA*	BAMA**	BAMA**
	Placebo	Vaccine	Placebo	Vaccine
gp70.A(92RW020)-V1V2	0/40 = 0%	118/246 = 48%	0.40 = 0%	178/246 = 72%
gp70.AE(92TH023)-V1V2	0/40 = 0%	217/246 = 88%	0/40 = 0%	237/246 = 96%
gp70.B(Case A2)-V1V2.LL	0/40 = 0%	204/246 = 83%	0/40 = 0%	178/246 = 72%
gp70.B(Case A2.p623)-V1V2.APorig	0/40 = 0%	168/246 = 68%	0/40 = 0%	138/246 = 56%
gp70.C(97ZA012)-V1V2	0/40 = 0%	187/246 = 76%	0/40 = 0%	142/246 = 58%
tags.C(1086)-V1V2	0/40 = 0%	210/246 = 85%	0/40 = 0%	234/246 = 95%

* For ELISA, the criterion for a positive response was a value of 3 SD above the mean of the Week 26 placebo group read-outs (n = 40) where read-outs are the natural logarithm-transformed averages of the OD readings across four replicates.

** For BAMA, a positive response is defined as (a) MFI greater than 263; (b) read-outs at Week 26 divided by read-outs at Week 0 greater than 3 for both blank-subtracted and blank-unsubtracted read-outs, and (c) blank MFI less than 5,000.

Since the original case-control analysis of the RV144 trial demonstrated that the IgA Env Ab level was a direct correlate of infection risk [3], we performed the primary statistical analysis with an “IgA-adjusted” model in order to provide the most sensitive analysis. **Table 4** shows the IgA-adjusted ORs and two-sided p-values indicating whether the ORs were significantly different from a value of one. The V1V2_orig_-gp70 and gp70.B(Case A2)-V1V2.LL, produced in different laboratories each gave essentially identical results (ORs = 0.62 and 0.60 for ELISA and ORs = 0.60 and 0.59 for BAMA, respectively), confirming and extending the results of the original RV144 correlates study (OR = 0.63; p = 0.018). Moreover, the results were similar in both of the immunoassays used with all of the antigens tested carrying V1V2 regions of different subtypes (A, B, C and AE) and with V1V2 regions presented in the context of distinct scaffolds. The level of Abs reactive with each of these antigens correlated inversely with the risk of HIV-1 infection (**Table 4**). These data demonstrate that cross-clade V1V2 IgG responses were elicited by immunization that each of these responses is a significant CoR, and that each of the six V1V2 antigens tested gave approximately the same correlation as the gp70_orig_-V1V2 variable (**Table 4** **)**.

**Figure 8:**
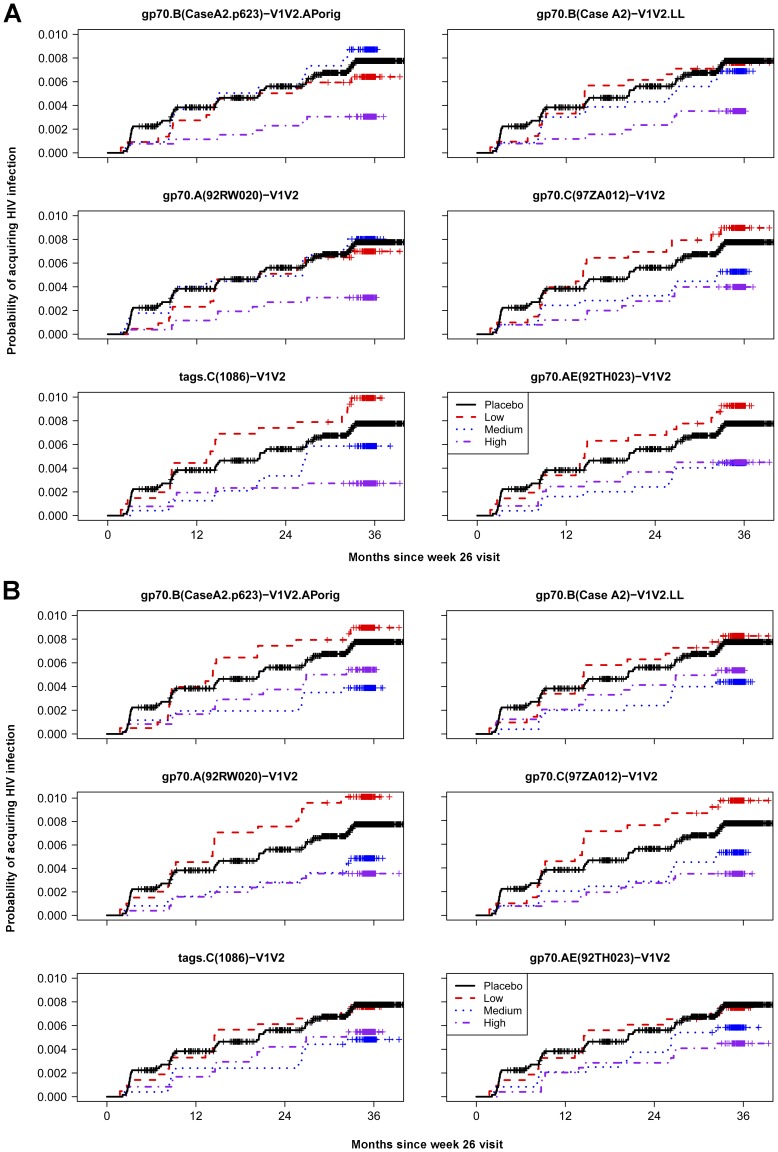
Estimated cumulative incidences of HIV-1 infection in placebo and vaccine recipients. The probability of acquiring HIV infection in vaccine recipients is shown for vaccine recipients with Low, Medium and High V1V2-scaffold IgG Ab responses as measured (A) by ELISA and (B) by BAMA at Week 26. The x-axis shows months since the Week 26 visit (two weeks post last immunization) and the y-axis shows the estimated probability of acquiring HIV-1 infection.

**Figure 9:**
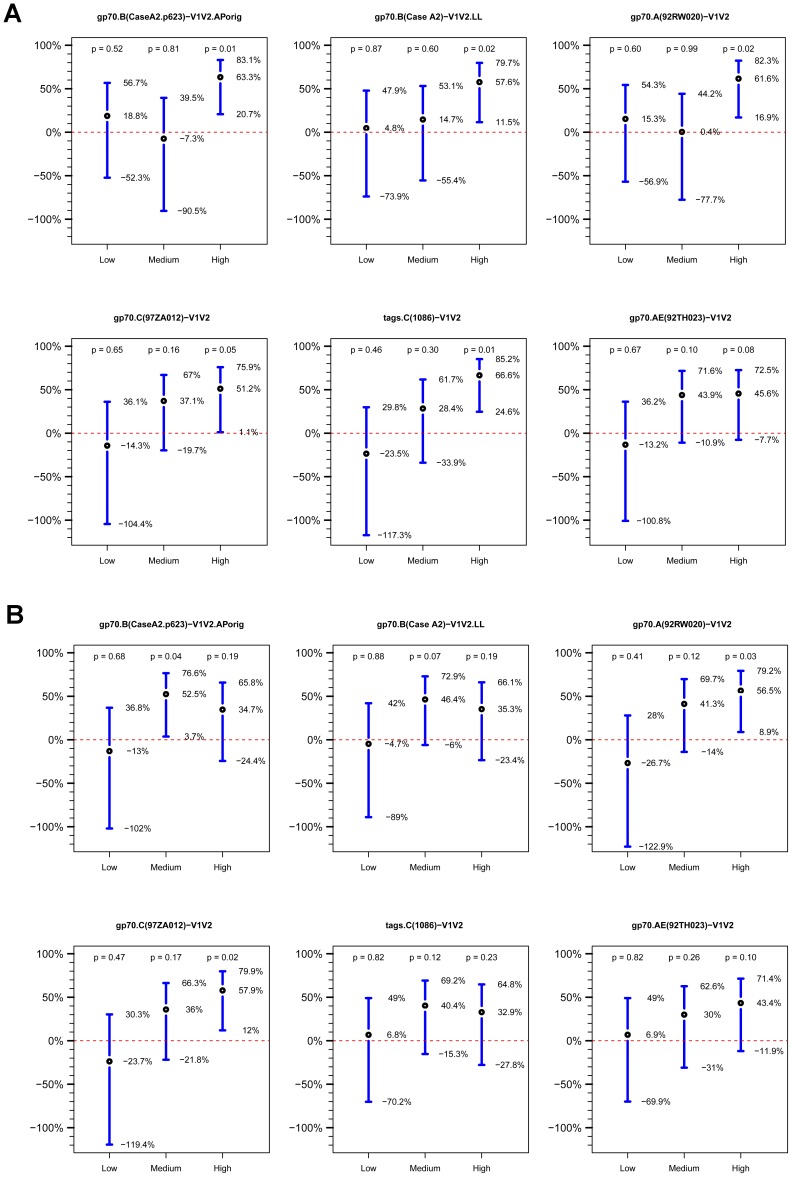
Estimated vaccine efficacy (VE). Estimated VE is shown for vaccine recipients with Low, Medium and High V1V2-scaffold IgG Ab responses at Week 26 versus the placebo group as defined by (A) ELISA data, and (B) BAMA data. VE is estimated as one minus the odds of infection in vaccine recipients with Low/Medium/High responses divided by the odds of infection in the entire HIV-1 negative placebo group at week 24. Points show the estimated VE values, and vertical lines delineate 95% confidence intervals. Two-sided p-values are from Wald tests.

The cumulative HIV-1 incidence categorized by the strength of the response to each antigen is shown in **Figure 8**. The estimated vaccine efficacy for Low, Medium and High responding vaccine subgroups versus the entire placebo group for each of the V1V2-scaffolds is shown in **Figure 9**. There is a trend suggesting a dose-response relationship with several of the antigens in both the ELISA and BAMA assay results, but these are not statistically significant.

The correlations of reactivities between pairs of antigens are shown in **Figure S1** (for ELISA) and **Figure S2** (for BAMA). The antigenic relationships of the V1V2-scaffold antigens based on BAMA results are shown in **Figure S3** and discussed in **Materials (File S1)**. To assess whether pairs of V1V2-scaffold reagents improved prediction of HIV-1 infection compared to individual reagents, reagent pairs with Spearman correlations below 0.90 were assessed in the same models. None of the reagent pairs significantly interacted in their prediction of infection, with the smallest q-value of 0.46 across the 27 interaction tests (for the 13 and 14 pairs assessed in the ELISA and BAMA, respectively) (**Tables S1 and S2 in File S1**; q-values are used as a technique for controlling the rate of false discovery results). In addition, none of the variable pairs were independent predictors as judged by both variables having p<0.10 in the same model, and, in general, the association of each reagent with infection risk was weakened by adding a second reagent (**Tables S1 and S2 in File S1**, and **S4 and S5**). Therefore, the results do not support that the different reagents measure Abs with different protective specificities.

Three of the four cross-reactivity score variables, which quantify the magnitude and breadth of reactivity across multiple subtype scaffolds (see **File S1**), were significant inverse CoRs of HIV-1 infection based on both ELISA and BAMA results (p-values <0.05), and the fourth score was a significant inverse CoR based on ELISA (p = 0.0078; p = 0.095 for BAMA) (**Table S3**). The ORs show that while cross-reactivity scores are highly predictive of infection risk, they do not predict risk better than the most predictive individual scaffolds.

## Discussion

Analysis of the immunologic reactivity of plasma specimens from the RV144 HIV-1 vaccine efficacy trial with a set of newly designed and synthesized V1V2-scaffold antigens demonstrates that the vaccine-induced Abs cross-react with the V1V2 regions from HIV-1 subtypes A, B, C and AE. In addition, the level of Abs cross-reactive with each of the V1V2 regions of these subtypes is a significant inverse CoR of HIV-1 infection. Notably, there was a significant inverse CoR provided by Ab reactivity with two antigens carrying V1V2 regions from subtype C: gp70.C(97ZA012)-V1V2 and tags.C(1086)-V1V2 derived from a transmitted/founder virus. Given that gp120 from the subtype C strain 1086 is intended for use as an immunogen in an upcoming HIV-1 vaccine efficacy trial in South Africa where subtype C predominates, the studies described here establish a link between the RV144 vaccine trial conducted in Thailand and the anticipated trial in South Africa.

Generation of cross-reactive protective Abs by active immunization is a key goal for vaccines designed to prevent infection with pathogens that exist in multiple and/or changing antigenic forms such as *Streptococcus pneumonia*, influenza, polio, dengue and malaria. A large body of data has now firmly established the cross-reactivity of Abs to the second and third variable regions of HIV-1, although “type-specific” variable loop Abs are also induced by the virus, particularly early after infection; moreover, cross-reactive V2 and V3 Abs mediate biologic functions such as neutralization against viruses from several subtypes of HIV-1 [2], . This cross-clade immunochemical and biologic activity suggests that variable regions might serve as biologically relevant vaccine targets, a hypothesis supported by the present study, the initial RV144 case-control study [3], and by analyses of breakthrough viruses and mAbs from the RV144 vaccine trial [9], [19].

The variable regions of the HIV-1 envelope are so named because of sequence changes between strains. Despite this sequence variation, these regions serve specific viral functions that constrain the sequence variation, and consequently the tertiary structures that these regions can assume. Thus, for example, while the sequence of the disulfide-linked V3 loop is highly variable, its length is essentially constant and approximately 65% of its amino acids are invariant or tolerate only conservative changes [20]. These constraints reflect the required participation of V3 in binding to cell surface co-receptors that mediate virus entry [21] and they permit anti-V3 Abs to cross-react with highly disparate V3 loops [22]. In contrast, V2 is more variable than V3, with greater variation in length and glycosylation [20], though some data support greater constraint of V2 length and glycosylation during the transmission bottleneck [23]–[25]. Nonetheless, many human mAbs that target V2 epitopes are known to recognize V2 regions from multiple subtypes [8], [9], [26], and RV144 vaccine-elicited V2-specific mAbs can mediate Tier 1 neutralization, ADCC, and the capture of infectious virions [9], [27]. These data suggest that antigenic and structural conservation within V2 underlies critical functions, a concept that is supported by bioinformatics data showing that V2 length may be a critical parameter for a functional envelope [20]. While V2 is similar to other variable regions in its flexible nature and propensity to flicker between conformations, it is hypothesized that it preferentially assumes lowest energy conformations which may represent the shapes that optimize biologic functions such as trimer stabilization [28], exposure of the α4β7 binding site which may facilitate binding to the surface of activated T cells (though this remains controversial [24], [29]), and protection of the CD4 and chemokine receptor binding sites on gp120 [28]. Thus, bioinformatics, structural, viral and immunologic studies converge to provide an explanation for Ab recognition of sequence-variable regions and suggest a means by which they may prevent infection by diverse HIV-1 strains.

Along with the data described above showing that highly cross-reactive vaccine-induced Abs reactive with V1V2-scaffold antigens predict a low risk of HIV-1 infection, there are additional published studies from the RV144 vaccine efficacy trial that identify V2 as a site targeted by vaccine-induced immune responses. Broad cross-reactivity of RV144 vaccinees’ plasma with linear and cyclic V2 peptides has previously been demonstrated [4], [30], and despite different methods and reagents, patterns of reactivity are similar to those documented above, e.g., strong reactivity with subtype AE V2 peptides and weak reactivity with subtype B. Sieve analysis of viral sequences isolated from vaccine and placebo recipients who became infected showed selective pressure on the virus at two positions in V2 [19], V2-specific polyfunctional effector memory CD4+ T cells were induced by the RV144 regimen [31], and V2-specific mAbs from RV144 vaccinees were shown to mediate various biologic functions [9]. Thus, several independent streams of data support the hypothesis that vaccine-induced, highly cross-reactive V1V2 Abs may have played a key role in reducing the infectivity of HIV-1. In addition, these data demonstrate that Abs specific for the V1V2 region of gp120 can be elicited with a relatively brief immunization regimen, and suggest that measurement of V1V2 Abs will provide an important endpoint to be evaluated in future testing of HIV-1 vaccine candidates.

## Supporting Information

Figure S1 **Spearman rank correlations and scatter plots based on ELISA data.** Data were generated with plasma from vaccine recipients drawn at Week 26 and tested against each of the six V1V2-scaffold antigens. The results shown in the first column and first row are those generated in the original case-control study [1].
(TIF)

Figure S2 **Spearman rank correlations and scatter plots based on BAMA data.** Data were generated with plasma from vaccine recipients drawn at Week 26 and tested against each of the V1V2-scaffold antigens.
(TIF)

Figure S3 **Antigenic map showing the relative positions of the 246 vaccine recipient case-control plasma samples.** Blue squares represent cases of infected vaccinees and red diamonds represent controls, uninfected vaccinees. Additionally, the positions in the antigenic map for the six new V1V2 scaffolds and the original V1V2-scaffold used in Haynes et al. [1], are denoted by text and circles. The map is based on six BAMA read-outs and is computed as described in **File S1**. The contour lines are based on ORs of HIV-1 infection computed over a grid of points within the map where the hypothetical read-out is based on distances within the map.
(TIF)

Figure S4 **Correlates of risk for pairs of scaffolds with data generated by ELISA.** Logistic regression models including gender, baseline behavioral risk, and IgA were conducted including all pairs of scaffolds with a pairwise Spearman correlation of less than 0.9. Pairs are ordered with the stronger (lower OR estimate in a model including only a single scaffold) correlate on the left. The pairs are ordered top to bottom by the change in the OR estimate of the stronger correlate between the two-scaffold model and the single-scaffold model. The arrows show the change in OR between the two-scaffold and single-scaffold models where the blue (red) arrow begins at the OR of the scaffold on the left (right) in the single-scaffold model and ends at the OR in the two-scaffold model. The direction of the arrow shows the direction of the change in ORs between the models. P-values for each scaffold in the two-scaffold model are shown on the left and right side. The Spearman rank correlation (r) between pairs is shown in parentheses. Interaction q-values are shown in parentheses and grayed out if above 0.20.
(TIF)

Figure S5 **Correlates of risk for pairs of scaffolds with data generated by BAMA.**
(TIF)

File S1 **Supplementary Appendix.** Statistical Analyses for the Study of the Reactivity of the Case-Control Plasma Panel from the RV144 Clinical HIV-1 Vaccine Trial with V1V2-scaffold Antigens.
(DOCX)

Protocol S1 **Clinical Study Protocol RV144.** A Phase III Trial of Aventis Pasteur Live Recombinant ALVAC-HIV (vCP1521) Priming With VaxGen gp120 B/E (AIDSVAX® B/E) Boosting in HIV-uninfected Thai Adults.
(PDF)
