# Supplementary figures and images for: Vaccine-Induced IgG Antibodies to V1V2 Regions of Multiple HIV-1 Subtypes Correlate with Decreased Risk of HIV-1 Infection
Source: PLoS One. 2014 Feb 4;9(2):e87572. doi: 10.1371/journal.pone.0087572 (PMC3913641; doi:10.1371/journal.pone.0087572)

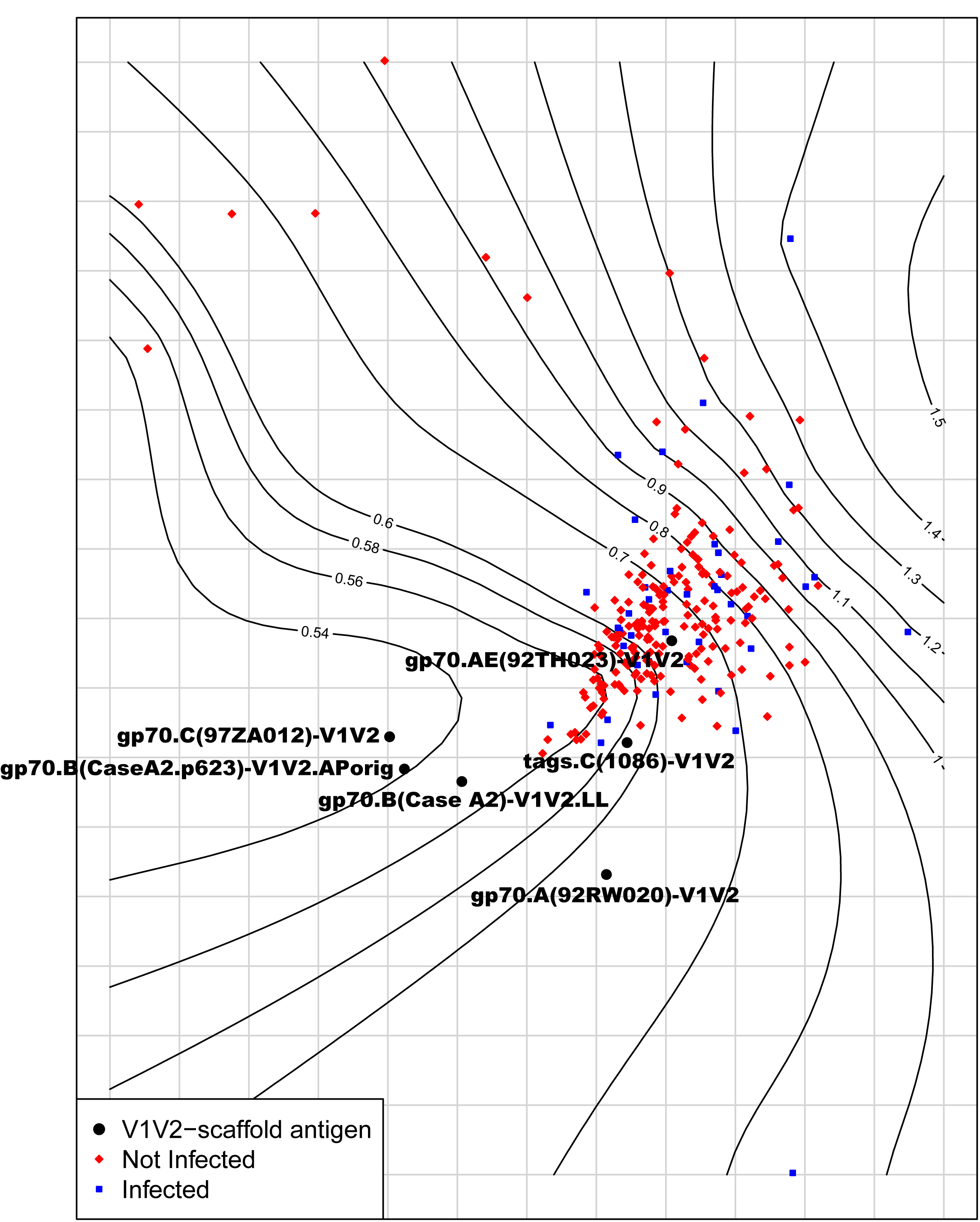

Supplement: Figure S3 — Antigenic map showing the relative positions of the 246 vaccine recipient case-control plasma samples. Blue squares represent cases of infected vaccinees and red diamonds represent controls, uninfected vaccinees. Additionally, the positions in the antigenic map for the six new V1V2 scaffolds and the original V1V2-scaffold used in Haynes et al. [1], are denoted by text and circles. The map is based on six BAMA read-outs and is computed as described in File S1. The contour lines are based on ORs of HIV-1 infection computed over a grid of points within the map where the hypothetical read-out is based on distances within the map. (TIF) [file pone.0087572.s003.tif]

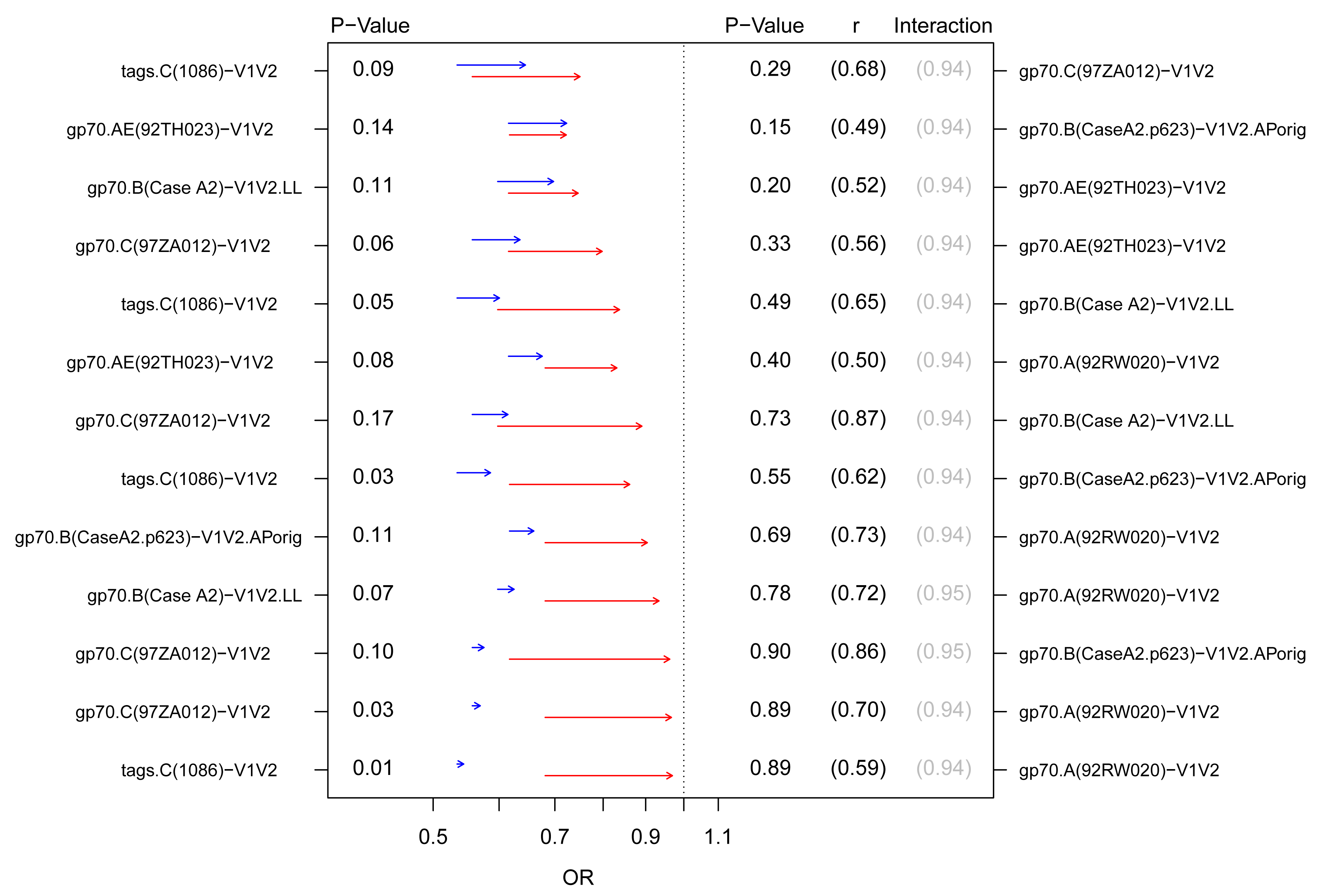

Supplement: Figure S4 — Correlates of risk for pairs of scaffolds with data generated by ELISA. Logistic regression models including gender, baseline behavioral risk, and IgA were conducted including all pairs of scaffolds with a pairwise Spearman correlation of less than 0.9. Pairs are ordered with the stronger (lower OR estimate in a model including only a single scaffold) correlate on the left. The pairs are ordered top to bottom by the change in the OR estimate of the stronger correlate between the two-scaffold model and the single-scaffold model. The arrows show the change in OR between the two-scaffold and single-scaffold models where the blue (red) arrow begins at the OR of the scaffold on the left (right) in the single-scaffold model and ends at the OR in the two-scaffold model. The direction of the arrow shows the direction of the change in ORs between the models. P-values for each scaffold in the two-scaffold model are shown on the left and right side. The Spearman rank correlation (r) between pairs is shown in parentheses. Interaction q-values are shown in parentheses and grayed out if above 0.20. (TIF) [file pone.0087572.s004.tif]

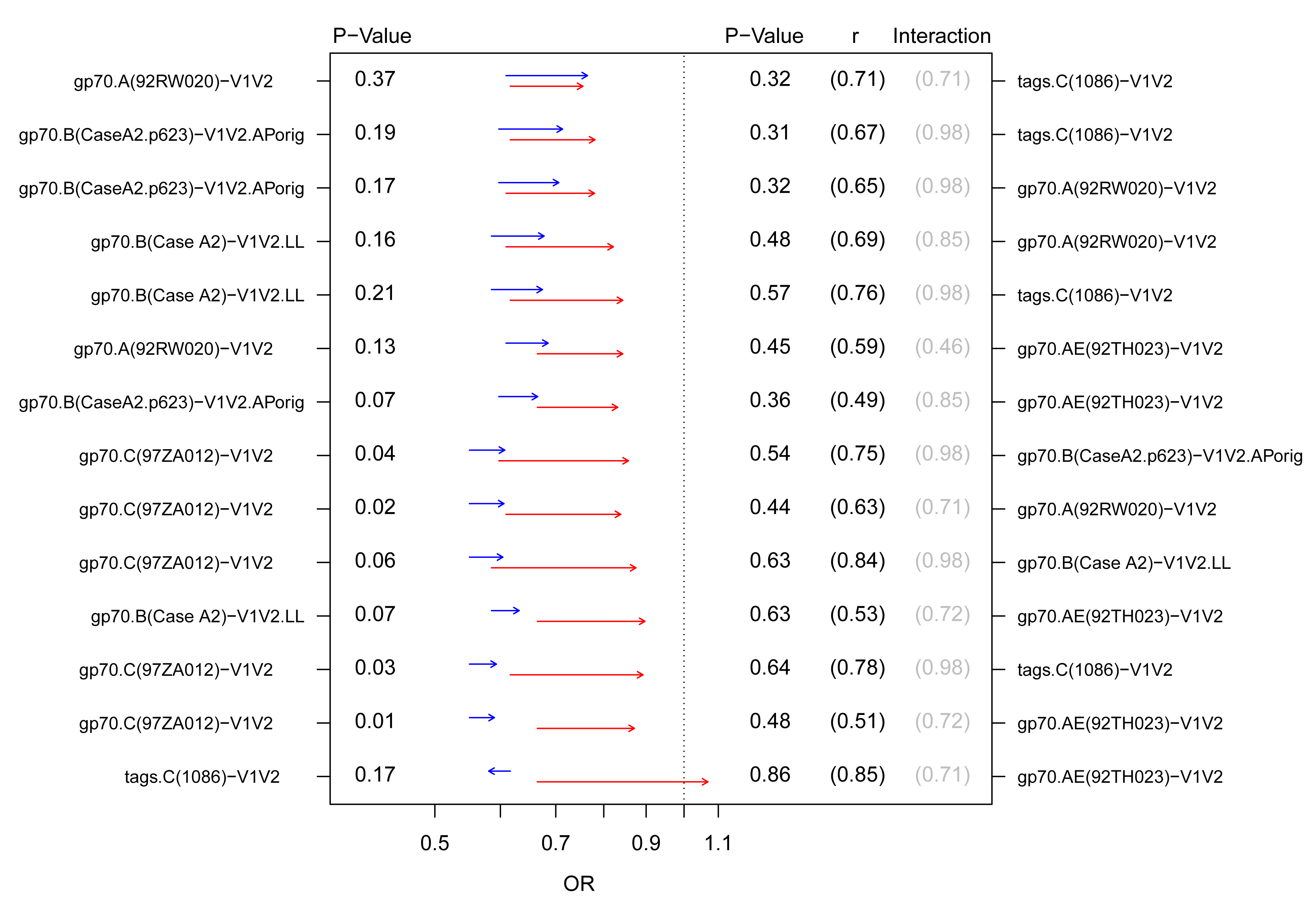

Supplement: Figure S5 — Correlates of risk for pairs of scaffolds with data generated by BAMA. (TIF) [file pone.0087572.s005.tif]
