# Supplementary material for: Vaccine-Induced IgG Antibodies to V1V2 Regions of Multiple HIV-1 Subtypes Correlate with Decreased Risk of HIV-1 Infection
Source: PLoS One. 2014 Feb 4;9(2):e87572. doi: 10.1371/journal.pone.0087572 (PMC3913641; doi:10.1371/journal.pone.0087572)
Supplement: File S1 — Supplementary Appendix. Statistical Analyses for the Study of the Reactivity of the Case-Control Plasma Panel from the RV144 Clinical HIV-1 Vaccine Trial with V1V2-scaffold Antigens. (DOCX) [file pone.0087572.s006.docx]

# ****Statistical Analyses for the Study of****

# ****the Reactivity of the Case-Control Plasma Panel from the****

# ****RV144 Clinical HIV-1 Vaccine Trial with V1V2-scaffold Antigens****

Supplementary Appendix to:

**Vaccine-induced IgG Antibodies to V1V2 Regions of Multiple HIV-1 Subtypes**

**Correlate with Decreased Risk of HIV-1 InfectioN**

Susan Zolla-Pazner^1,2^, Allan deCamp^3^, Peter B. Gilbert^3^, Constance Williams^2^, Nicole L Yates^4^ William T. Williams^4^, Robert Howington^4^, Youyi Fong^3^, Daryl E. Morris^3^, Kelly A. Soderberg^4^, Carmela Irene^5^, Charles Reichman^5^, Abraham Pinter^5^, Robert Parks^4^, Punnee Pitisuttithum^6^, Jaranit Kaewkungwal^6^, Supachai Rerks-Ngarm^7^, Sorachai Nitayaphan^8^, Charla Andrews^9^, Robert J O'Connell^9^, Zhi-yong Yang^10^, Gary J. Nabel^10§^, Jerome H. Kim^9^, Nelson L. Michael^9^, David C. Montefiori^4^, Hua-Xin Liao^4^, Barton F. Haynes^4^, and Georgia D. Tomaras^4^.

^1^Department of Veterans Affairs New York Harbor Healthcare System, ^2^NYU School of Medicine, New York, NY; ^3^Vaccine and Infectious Disease Division,^,^ Fred Hutchinson Cancer Research Center, Seattle, WA; ^4^Duke University, Durham, NC; ^5^Public Health Research Institute, University of Medicine and Dentistry, Newark, NJ; ^6^Faculty of Tropical Medicine, Mahidol, Thailand; ^7^Ministry of Public Health, Thailand; ^8^AFRIMS, Bangkok, Thailand; ^9^Military HIV Research Program, Walter Reed Army Institute of Research, Silver Spring, MD; ^10^Virology Laboratory, Vaccine Research Center, National Institute for Allergy and Infectious Diseases, National Institutes of Health, Bethesda, MD.

^§^Current address: Sanofi Aventis U.S. LLC, 270 Albany St, Cambridge, MA 02139

**Statistical analyses**

A primary criterion for down-selecting scaffold antigens to the case-control study was the Spearman rank correlation between Week 26 vaccine recipient read-outs of the new scaffold antigens and the original V1V2 antigen used for the RV144 correlates analysis [[1](#_ENREF_1)]. Antigens highly correlated with the original antigen would be unlikely to result in meaningful differences in a repeated correlates analysis. Nevertheless, we selected some antigens that were highly correlated with the original antigen in order to definitively replicate the original V1V2 correlate of risk (CoR) result. Spearman rank correlations between all pairs of antigens were also assessed as a down-selection criterion (with the purpose to remove redundant antigens and maximize the diversity of V1V2 responses) and for informing multivariate CoR modeling that assesses multiple scaffold antigens in the same regression model.

Heat maps were used to graphically display the Spearman rank correlations. Hierarchical clustering trees using complete linkage based on a distance measure defined as one minus the Spearman rank correlation coefficient between Week 26 read-outs of vaccine recipients were used to cluster antigens into common antigenic groups. The choice of the number of clusters was informed by the target number of antigens (6 to 10) that would be advanced to the next phase of the down-selection process. When selecting one or more antigens within a cluster we used the following additional criteria for selection: 1) the estimated Week 26 signal-to-noise ratio defined as the standard deviation of the subject-specific read-outs divided by the mean of the standard deviation of the within-subject replicates; 2) the Week 26 positive response rates among vaccine recipients, defined using a cutoff of 3 standard deviations above the mean of the Week 0 read-outs for a given antigen; 3) the inter-quartile ranges of each read-out; and 4) the p-value from a Wilcoxon rank sum test comparing the distribution of vaccine and placebo recipient read-outs. In the Phase 1 pilot study, an antigenic map of the scaffold antigens and Week 26 plasma samples was constructed by the method of Multidimensional Scaling (MDS) using the SMACOF library in R [[2](#_ENREF_2)] and angles between antigens within the map were used as an additional selection criterion. Angles between pairs of antigens were computed using the medoid of the mapped plasma coordinates as a vertex [[3](#_ENREF_3)].

In addition to the individual antigen read-outs, four immune score variables measuring cross-reactivity, three categorical and one quantitative, were assessed as CoRs for both ELISA and BAMA. Among the six V1V2-scaffold antigens, two were derived from subtype B strains, two were derived from subtype C strains, one was derived from a subtype A strain, and one was derived from a subtype CRF01_AE strain. To define the categorical score variables we first set a response threshold at the 33^rd^ (or 50^th^, or 66^th^) percentile of the vaccine group response. A count of the number of distinct subtypes with a reagent reaction above this threshold was computed for each subject, and these counts were categorized as Low, Medium and High such that each group had approximately equal numbers (pooling over cases and controls). To define the quantitative score variable we summed the scaled (mean zero and standard deviation one) read-outs across the four subtypes where the maximum of the two subtype B read-outs, and similarly the two subtype C read-outs, was first computed before adding the read-outs of the subtype A and AE antigens.

For the case-control analysis an antigenic map of the six V1V2-scaffold antigens and the 246 vaccine group plasma samples was computed using MDS. Antigenic maps have been used previously to visualize binding assay data from influenza [[4](#_ENREF_4)] [[5](#_ENREF_5)]. An antigenic map assigns each antigen and plasma sample a point in a low dimensional Euclidean space such that the distance between the antigen and plasma sample in the map is approximated by a specific transformation of the original assay read-out. We generated a map in two dimensions based on the following transformation:

$$\log\left( \mathrm{MFI} \right)=\log\left( 32000 \right)-d$$

where $d$ is the distance in the map and log is the natural logarithm. The value 32000 is the highest possible read-out in BAMA. Therefore, distance between V1V2-scaffold antigens and plasma samples approximate the original natural logarithm transformed BAMA read-out through a simple linear relationship. Contour lines are estimated ORs computed over a grid of points within the map where a hypothetical read-out is computed for each grid point based on the distances between that point and the plasma samples within the map. Although we do not know where additional V1V2-scaffold antigens would fall within the map had we run them in the assay, the contour lines allow us to see the effect of changes in location within the map on the estimated OR of HIV-1 infection were we able to measure additional read-outs. Additionally, even though there may not exist a V1V2-scaffold antigen that occupies every point on the map, we can use the contour lines to derive a theoretical location within the map that best discriminates risk of infection.

**Interpretation of antigenic map derived from bamA read-outs.**

Based on subtype match, we assume that the gp70.AE(92TH023)-V1V2 is antigenically most similar to the majority of circulating strains in the trial population. From the contour lines of the antigenic map (**Supplemental Figure S3**), we can see that when probing plasma responses with different V1V2-scaffold antigens, the strongest predictor of infection risk was not the antigen most closely related to the circulating strains (here represented by gp70.AE(92TH023)-V1V2) but rather an antigen that carried non-clade AE V1V2 regions (here represented by gp70.C(97ZA012)-V1V2 and gp70.B(Case A2.p623)-V1V2.AP_orig_). We hypothesize that antigenic maps can inform decisions regarding which candidate vaccine immunogen(s) should be advanced to efficacy trials and which antigens are best to use to determine significant correlates of risk.

**REFERENCES**

**1. Haynes BF, Gilbert PB, McElrath MJ, Zolla-Pazner S, Tomaras GD, et al. (2012) Immune Correlates Analysis of the ALVAC-AIDSVAX HIV-1 Vaccine Efficacy Trial. N Engl J Med 366: 1275-1286.**

**2. De Leeuw J, Mair P (2009) Multidimensional scaling using majorization: SMACOF in R. Journal of Statistical Software 31: 1-30.**

**3. Kaufman L, Rousseeuw PJ (1990) Finding groups in data: an introduction to cluster analysis: Wiley, NY.**

**4. Lapedes A, Farber R (2001) The geometry of shape space: application to influenza. J Theor Biol 212: 57-69.**

**5. Smith DJ, Lapedes AS, de Jong JC, Bestebroer TM, Rimmelzwaan GF, et al. (2004) Mapping the antigenic and genetic evolution of influenza virus. Science 305: 371-376.**

**Supplemental Table S1.** Estimated odds ratios and p-values for models based on reagent pairs in ELISA*

|  |  | **Corr.** | **Scaffold 1** | | **Scaffold 2** | | **Interaction** |
| --- | --- | --- | --- | --- | --- | --- | --- |
| **Scaffold 1** | **Scaffold 2** | **r** | **OR** | **p-value** | **OR** | **p-value** | **q-value** |
| gp70.B(CaseA2.p623)-V1V2.APorig | gp70.B(Case A2)-V1V2.LL | 0.97 | NA | | | | |
|  | gp70.A(92RW020)-V1V2 | 0.73 | 0.66 | (0.11) | 0.90 | (0.69) | 0.94 |
|  | gp70.C(97ZA012)-V1V2 | 0.86 | 0.96 | (0.90) | 0.58 | (0.10) | 0.95 |
|  | tags.C(1086)-V1V2 | 0.62 | 0.86 | (0.55) | 0.59 | (0.03) | 0.94 |
|  | gp70.AE(92TH023)-V1V2 | 0.49 | 0.72 | (0.15) | 0.72 | (0.14) | 0.94 |
| gp70.B(Case A2)-V1V2 | gp70.A(92RW020)-V1V2 | 0.72 | 0.63 | (0.07) | 0.93 | (0.78) | 0.95 |
|  | gp70.C(97ZA012)-V1V2 | 0.87 | 0.89 | (0.73) | 0.61 | (0.17) | 0.94 |
|  | tags.C(1086)-V1V2 | 0.65 | 0.84 | (0.49) | 0.60 | (0.05) | 0.94 |
|  | gp70.AE(92TH023)-V1V2 | 0.52 | 0.70 | (0.11) | 0.75 | (0.20) | 0.94 |
| gp70.A(92RW020)-V1V2 | gp70.C(97ZA012)-V1V2 | 0.70 | 0.97 | (0.89) | 0.57 | (0.03) | 0.94 |
|  | tags.C(1086)-V1V2 | 0.59 | 0.97 | (0.89) | 0.54 | (0.01) | 0.94 |
|  | gp70.AE(92TH023)-V1V2 | 0.50 | 0.83 | (0.40) | 0.68 | (0.08) | 0.94 |
| gp70.C(97ZA012)-V1V2 | tags.C(1086)-V1V2 | 0.68 | 0.75 | (0.29) | 0.65 | (0.09) | 0.94 |
|  | gp70.AE(92TH023)-V1V2 | 0.56 | 0.64 | (0.06) | 0.80 | (0.33) | 0.94 |
| tags.C(1086)-V1V2 | gp70.AE(92TH023)-V1V2 | 0.91 | NA | | | | |

* For each pair an interaction q-value is also reported. ORs and p-values as well as the interaction q-value are reported only for reagent pairs with Spearman rank correlations (r) below 0.90.

**Supplemental Table S2.** Estimated odds ratios and p-values for models based on reagent pairs in BAMA*

|  |  | **Corr.** | **Scaffold 1** | | **Scaffold 2** | | **Interaction** |
| --- | --- | --- | --- | --- | --- | --- | --- |
| **Scaffold 1** | **Scaffold 2** | **r** | **OR** | **p-value** | **OR** | **p-value** | **q-value** |
| gp70.B(CaseA2.p623)-V1V2.APorig | gp70.B(Case A2)-V1V2.LL | 0.97 | NA | | | | |
|  | gp70.A(92RW020)-V1V2 | 0.65 | 0.71 | (0.17) | 0.78 | (0.32) | 0.98 |
|  | gp70.C(97ZA012)-V1V2 | 0.75 | 0.86 | (0.54) | 0.61 | (0.04) | 0.98 |
|  | tags.C(1086)-V1V2 | 0.67 | 0.71 | (0.19) | 0.78 | (0.31) | 0.98 |
|  | gp70.AE(92TH023)-V1V2 | 0.49 | 0.67 | (0.07) | 0.83 | (0.36) | 0.85 |
| gp70.B(Case A2)-V1V2.LL | gp70.A(92RW020)-V1V2 | 0.69 | 0.68 | (0.16) | 0.82 | (0.48) | 0.85 |
|  | gp70.C(97ZA012)-V1V2 | 0.84 | 0.87 | (0.63) | 0.60 | (0.06) | 0.98 |
|  | tags.C(1086)-V1V2 | 0.76 | 0.67 | (0.21) | 0.84 | (0.57) | 0.98 |
|  | gp70.AE(92TH023)-V1V2 | 0.53 | 0.63 | (0.07) | 0.90 | (0.63) | 0.72 |
| gp70.A(92RW020)-V1V2 | gp70.C(97ZA012)-V1V2 | 0.63 | 0.84 | (0.44) | 0.61 | (0.02) | 0.71 |
|  | tags.C(1086)-V1V2 | 0.71 | 0.76 | (0.37) | 0.75 | (0.32) | 0.71 |
|  | gp70.AE(92TH023)-V1V2 | 0.59 | 0.69 | (0.13) | 0.84 | (0.45) | 0.46 |
| gp70.C(97ZA012)-V1V2 | tags.C(1086)-V1V2 | 0.78 | 0.59 | (0.03) | 0.89 | (0.64) | 0.98 |
|  | gp70.AE(92TH023)-V1V2 | 0.51 | 0.59 | (0.01) | 0.87 | (0.48) | 0.72 |
| tags.C(1086)-V1V2 | gp70.AE(92TH023)-V1V2 | 0.85 | 0.58 | (0.17) | 1.07 | (0.86) | 0.71 |

* For each pair an interaction q-value is also reported. ORs and p-values as well as the interaction q-value are reported only for reagent pairs with Spearman rank correlations r below 0.90.

**Supplemental Table S3.** Estimated odds ratios and p-values comparing High versus Low (upper and lower third responses) vaccine recipient subgroups for the six V1V2-scaffold antigens and the three cross-reactivity score variables based on ELISA and BAMA read-outs*.

|  | **ELISA** | | **BAMA** | |
| --- | --- | --- | --- | --- |
| **V1V2 Scaffold Antigen** | **OR** | **p-value** | **OR** | **p-value** |
| gp70.B(CaseA2.p623)-V1V2.AP_orig_ | 0.37 | 0.041 | 0.40 | 0.039 |
| gp70.B(Case A2)-V1V2.LL | 0.33 | 0.020 | 0.45 | 0.072 |
| gp70.A(92RW020)-V1V2 | 0.32 | 0.023 | 0.21 | 0.0015 |
| gp70.C(97ZA012)-V1V2 | 0.31 | 0.012 | 0.25 | 0.004 |
| tags.C(1086)-V1V2 | 0.22 | 0.0019 | 0.55 | 0.170 |
| gp70.AE(92TH023)-V1V2 | 0.41 | 0.037 | 0.49 | 0.110 |
| Score 1 [No. subtypes > 33^rd^ percentile] | 0.42 | 0.037 | 0.43 | 0.044 |
| Score 2 [No. subtypes > 50^th^ percentile] | 0.31 | 0.019 | 0.30 | 0.017 |
| Score 3 [No. subtypes > 67^th^ percentile] | 0.29 | 0.0078 | 0.46 | 0.095 |

*The three cross-reactivity score variables and the High and Low categories for these variables are described in **Supplementary Methods**. Briefly, the scores count the number of distinct subtypes with a reagent reaction above the 33^rd^, 50^th^, or 66^th^ percentile of the vaccine group response, and High and Low are the upper and lower tertiles of these counts in the vaccine group.
